# An Enhanced Tree Routing Based on Reinforcement Learning in Wireless Sensor Networks †

**DOI:** 10.3390/s23010223

**Published:** 2022-12-26

**Authors:** Beom-Su Kim, Beomkyu Suh, In Jin Seo, Han Byul Lee, Ji Seon Gong, Ki-Il Kim

**Affiliations:** 1 Department of Computer Science and Engineering, Chungnam National University, Daejeon 34134, Republic of Korea; 2 Smart Power Distribution Lab, Korea Electric Power Research Institute, Daejeon 34056, Republic of Korea

**Keywords:** wireless sensor networks (WSNs), tree-based routing, reinforcement learning, Q-learning, multiple objectives

## Abstract

In wireless sensor networks, tree-based routing can achieve a low control overhead and high responsiveness by eliminating the path search and avoiding the use of extensive broadcast messages. However, existing approaches face difficulty in finding an optimal parent node, owing to conflicting performance metrics such as reliability, latency, and energy efficiency. To strike a balance between these multiple objectives, in this paper, we revisit a classic problem of finding an optimal parent node in a tree topology. Our key idea is to find the best parent node by utilizing empirical data about the network obtained through Q-learning. Specifically, we define a state space, action set, and reward function using multiple cognitive metrics, and then find the best parent node through trial and error. Simulation results demonstrate that the proposed solution can achieve better performance regarding end-to-end delay, packet delivery ratio, and energy consumption compared with existing approaches.

## 1. Introduction

A wireless sensor network (WSN) enables the automation of industrial sites through the collection, exchange, and analysis of data between interconnected sensor devices. One of the promising WSN applications is a real-time monitoring system in which sensor nodes collect data and forward them to a sink node through a multi-hop transmission. For the freshness of data, the monitoring system has a strong demand for real-time communication; hence, it is important to design a low-latency and highly reliable routing protocol. Specifically, a routing protocol for monitoring applications has the following requirements:

(a) High reliability: Considering the limited transmission range of sensors, sampled data should be forwarded to the destination through a multi-hop transmission after self-configuration of a network. However, if data are frequently dropped owing to poor link quality between sensors, the service requirements, especially for mission-critical applications, cannot be satisfied.

(b) Low latency: The data sampled from sensors should be delivered to the destination quickly; otherwise, it will not be possible to respond to emergencies. In particular, stale data are fatal for patients with serious illness in a health monitoring system. Hence, it is necessary to build the shortest path or reduce the queuing delay via load balancing to forward the data to the destination in a timely manner.

(c) Energy efficiency: Sensors for monitoring applications are typically installed in a wild field where battery replacement is difficult. If the energy of a specific node is quickly exhausted, it leads to a decrease in the network lifetime. Thus, it is necessary to reduce the additional energy consumption owing to network maintenance or to balance energy consumption between sensors.

Conventional ad hoc routing protocols [1,2] can ensure reliable transmission and satisfy service requirements of source nodes in WSNs; however, they involve a high computational cost (i.e., control-message overhead) for sensors to maintain routing paths. To reduce the energy consumption from the control overhead, tree-based routing can be a feasible solution in WSNs. The parent selection is a key role of a tree-based routing protocol because the links between parent and child nodes are used as routing paths. Conventional tree routing [3] uses the number of hops to the sink as a decision criterion for parent selection. Each source node can build the shortest path to the sink by choosing a node with the least number of hops as a parent node. This approach can provide a low latency; however, it cannot ensure reliable transmission because the link quality between the parent node and the child node is not considered in the parent selection process.

To build a stable tree topology, some studies [4,5] adopt the link quality as a decision criterion. However, they do not consider the load balancing during the parent selection; hence, an excessive load can be placed on a specific node, resulting in significant packet loss. To prevent congestion, some studies [6,7,8] propose a load-aware parent selection algorithm; however, they do not jointly consider link quality and energy efficiency. Although energy-aware parent selection schemes are proposed [9,10,11,12,13], they also cannot jointly achieve the above service requirements. These tree-based routing protocols address diverse parent selection problems independently; hence, they cannot jointly improve multiple performance metrics, such as the transmission reliability, end-to-end delay, and energy consumption.

To address this problem, some studies [14,15,16,17,18] attempt to select the best parent node taking into account multiple cognitive metrics. To jointly achieve multiple objectives, they employ a linear weighted-sum method in which each node calculates the weighted cost by integrating multiple metrics. However, the linear weighted-sum method adopts subjective weights, which are often less objective and reduce their acceptance within the scientific community. As a result, they cannot provide a flexible trade-off between performance metrics. To overcome this limitation, in our previous work [19], we proposed a multi-criteria decision making (MCDM)-based parent selection scheme. The proposed scheme finds the best parent node by determining the relative importance of multiple decision criteria using the analytical hierarchy process (AHP) [20] and then derives the weighted cost using the simple additive weighting (SAW) method [21]. However, the decision maker needs to calculate the weights of each metric based on its preference or background knowledge of the target network. In addition, it is challenging to find an optimal parent node in a large-scale WSN because the scale of WSNs is growing and the deployment is more complex.

To eliminate the bias of a decision maker and make adaptive decisions in a complex deployment scenario, in this work, we extend our previous work with the specific goal of achieving multiple objectives, such as high reliability, low latency, and energy efficiency. To find the best parent node, we use empirical data about the target network obtained through Q-learning. Specifically, we define a state space, action set, and reward function using multiple cognitive metrics, and then find the best parent node through trial and error.

The main contributions of this work are summarized as follows:

- To jointly achieve multiple objectives in WSNs, in this work, we revisit the classic problem of finding an optimal parent node in the tree-based routing. Specifically, we propose an enhanced tree-based routing protocol based on reinforcement learning (RL);
- To cope with various network scenarios, e.g., link breakage and congestion, we propose multiple cognitive metrics. Specifically, in addition to hop count, three types of cognitive metrics are formulated: weighted average of received signal strength, buffer occupancy ratio, and power consumption ratio. These metrics affect the decision rules in tree routing;
- We present the system model for applying RL algorithm in WSNs and specify the basic operations of the proposed tree-based routing protocol. Specifically, we specify concrete algorithms for loop detection and parent update, as well as tree construction using build and hello messages;
- To make adaptive decisions in a complex deployment scenario, we define a state space, action set, and reward function. The agent recognizes the network state with the proposed cognitive metrics and finds the best parent node with the highest reward through trial and error;
- Through a comparative study using diverse simulations, we verify that the proposed parent selection scheme supports a reasonable trade-off between the performance metrics, i.e., end-to-end delay, packet delivery ratio, and energy consumption.

## 2. Related Work

The routing protocol has an important role in a WSN, ensuring a reliable transmission and satisfying the QoS of individual nodes. The node batteries of a network in the wild are difficult to replace; hence, the routing protocols should create a routing path at low computational cost. In reactive routing protocols, e.g., ad hoc on-demand distance vector (AODV) routing [1], a source node sends a route request message whenever it needs a routing path to the destination node. To maintain a reliable routing path, the node periodically broadcasts control messages and checks the status of the links with its neighboring nodes. Reactive routing can guarantee a reliable transmission in a network in which the nodes frequently move, such as mobile ad hoc networks; however, the control overhead is significantly increased to maintain the routing paths.

To reduce the number of control messages, proactive routing protocols, e.g., optimized link state routing protocol [2], select nodes with high connectivity between neighboring nodes as the parent nodes. To maintain the topology information, the parent nodes are obliged to broadcast control messages periodically. This table-driven routing can minimize the number of control messages because only the parent nodes broadcast the topology information; however, the broadcast overhead and memory usage are increased when there is a large amount of node information to be transmitted. Moreover, in a network in which the nodes frequently move, the control overhead significantly increases because topology information must be frequently broadcast. To reduce the energy consumption from the broadcast overhead, tree-based routing is the best solution for a small-scale WSN. A tree-based routing protocol consists of one root node and multiple sensor nodes, which have parent–child relationships and are interconnected based on a tree structure. Each node chooses the optimal parent node and forwards the data to the parent until it reaches the root node. Tree-based routing can eliminate a path search and avoid extensive broadcast messages. The communication links between parent and child nodes are used as routing paths, and parent selection is a key aspect of the tree-based routing protocol.

For example, conventional tree-based routing protocols [22,23,24] select as the parent node the neighbor node with the lowest number of hops to the sink node. By using the hop count as a decision metric, they can minimize the end-to-end delay; however, the PDR is reduced because frequent node movements degrade the link quality between the child and parent nodes. To build a stable tree structure, the authors of [4] propose a route stability framework in which each node calculates an overall score for each neighboring node. The scores are weighted based on the link quality and relative distance with the neighboring node. The neighbor node with the best overall score is selected as a parent node. In [5], each node chooses the neighboring node with the best link quality metric as a parent node. The link quality metric is calculated using cognitive metrics, i.e., the link packet delivery and link stability. However, this approach does not consider the load balancing during the parent selection process. In other words, an excessive load may be placed on a particular node; thus, congestion occurs at certain nodes, resulting in significant packet loss. In addition, the network lifetime is shortened due to unbalanced energy consumption between nodes.

To prevent congestion, the authors of [6] propose a congestion-aware tree routing protocol. With this scheme, each node evaluates the link cost of each neighboring node, and the node with the best link cost is selected. A weighted-sum equation is used to determine the link cost, and the average queue size and remaining energy of the nodes are considered in the proposed formula. The authors of [7] propose a load-aware dynamic cost function to weight the links between the parent and child nodes. The cost of the links is updated based on the congestion metric. If a node is congested, the value of congestion metric will increase. In this case, it is not preferable for neighboring nodes to select this link as a parent node. To improve the network lifetime, the authors of [9] propose an energy-aware parent node selection algorithm. With this scheme, each node builds a hierarchical structure based on the hop count to the sink node. If the energy level of a node is lower than half of the original battery capacity, the node is moved one level lower in the tree model. By lowering the hierarchical level, a node with a low battery level cannot be selected as a parent node. The authors of [11] construct a binary tree based on the number of hops to the sink node. To balance the energy consumption, the parent node chooses the link with the smaller number of packets it receives from the child on the left or right. In [10,12], each node calculates the link cost using the distance vector and remaining energy. In [13], each node selects a parent node from the neighboring nodes with predefined cognitive parameters, which is called a fitness function using the flower pollination algorithm. The fitness function considers the energy consumption and distance ratios of the average distance.

Several tree-based routing protocols take multiple cognitive metrics into consideration concurrently and aim to jointly achieve multiple objectives by considering several decision criteria. For example, the authors of [14] take three cognitive metrics, i.e., the channel error rate, residual energy, and buffer capacity, and integrate the proposed metrics using heuristic coefficients. These coefficients denote the significance of each metric, and the sum of the coefficients is equal to 1. Each node then selects the neighboring node with the highest weighted sum as a parent node. Similarly, the authors of [15] take the link quality, residual energy, and relay node frequency and integrate these metrics using the weighted-sum method. The authors of [16] integrate multiple cognitive metrics, namely, the distance, number of associated nodes, and residual energy using the weighted-sum method. The authors of [17] integrate three cognitive metrics, such as the hop count, residual energy, and link quality using the heuristic coefficients. The authors of [18] consider four attributes, i.e., the link expiration time, trip time, node speed, and lifetime, and integrate them using an “if-else” statement. However, these approaches adopt subjective weights, which are often less objective and reduce their acceptance by the scientific community. To provide a flexible trade-off between the conflicting objectives, in our previous work [19], we propose a MCDM-based parent selection scheme in which each node finds the best parent node by determining the relative importance of multiple decision criteria using the AHP and then derives the weighted cost using the SAW method.

## 3. System Model and Problem Statement

### 3.1. Network Model

**Figure 1:**
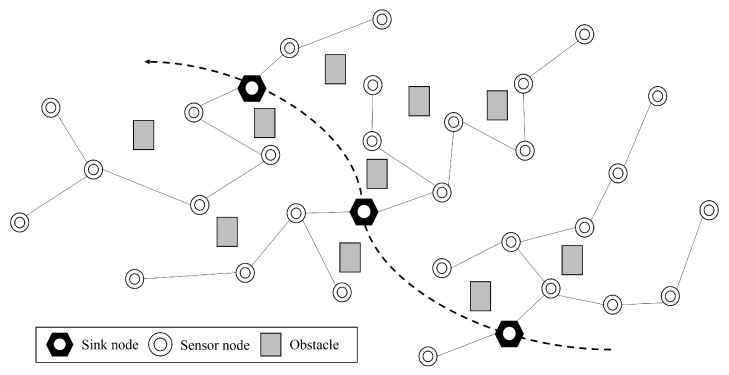
An example: deployment of sink nodes and sensor nodes.

As shown in Figure 1, there are *N* sensor nodes and *M* sink nodes. These nodes constitute a tree topology in which the sink node is located at the top level of the tree. The tree structure is autonomously configured via a tree routing protocol (see Section 4). The sink node acts as a controller to aggregate the data sampled from the sensor nodes and forms a tree structure. We assume that both the sensor node and the sink node have fixed positions and the link quality between nodes depends on geographical positions. Each sensor node then selects a parent node among its neighbors and passes the data to the parent node until it reaches a sink node.

### 3.2. Cognitive Metrics

To select an optimal parent node according to changes in network conditions, each node needs to recognize the current network state using multiple cognitive metrics. Recall that the main goal of this study is to jointly achieve the high reliability, low latency, and energy efficiency. However, there are many considerations to select the best parent node, for example, in a scenario where multiple sensor nodes choose the same parent, a queue is building at the parent node and thus the queueing delays increase or packets are lost. In addition, the link quality between the parent node and the child node depends on their geographical positions; hence, obstacles can make the transmission unreliable. To address diverse parent selection problems, we define the following cognitive metrics.

#### 3.2.1. Hop Count

To reduce the latency for each transmission, each node should build a shortest path to the destination. In a tree-based routing, the number of hops to the sink node is a decisive metric to select a parent node; that is, each node can maintain a low latency by adopting the hop count to the sink node as a decision variable. In this work, we adopt the hop count (*H*) as a decision variable to form a hierarchical tree structure and reduce the end-to-end delay.

#### 3.2.2. Weighted Average of Received Signal Strength

The link quality between the child node and the parent node is highly dependent on distance and obstacles. If a node chooses the node with the shortest hop to the sink node as a parent node but the link quality is poor, packets may be lost and additional energy consumption may increase owing to retransmission. To build a stable path, we use the received signal strength between the child node and the candidate parent node. Each node can easily obtain the received signal strength when it receives a hello message from neighboring nodes.

Contrary to the cognitive metrics proposed in our previous study, we use weighted moving average (WMA) to prevent sudden changes in measured values. The WMA gives more weights on recent data and less on past data. That is, we multiply each measured value by a weight. Here, the time *t* is based on the time of a hello message. We define a *n*-period WMA of the received signal strength (*P*) for neighboring node *i* as follows:

(1) $$P_{i}=\frac{\left(w_{1}*P_{i}^{t-1}+w_{2}*P_{i}^{t-2},\ldots ,+w_{n}*P_{i}^{t-n}\right)}{\left(w_{1}+w_{2},\ldots ,+w_{n}\right)},$$

#### 3.2.3. Weighted Average of Buffer Occupancy Ratio

If multiple nodes select the same neighbor as a parent node, the selected parent node will be overloaded. In other words, a buffer overflow may occur when the parent node receives more data than it can handle. To prevent congestion, we adopt the buffer occupancy ratio as a decision variable to select a parent node. To prevent sudden changes in measured data, we define a *n*-period WMA of the buffer occupancy ratio (*B*) for neighboring node *i* as,

(2) $$B_{i}=\frac{\left(w_{1}*B_{i}^{t-1}+w_{2}*B_{i}^{t-2},\ldots ,+w_{n}*B_{i}^{t-n}\right)}{\left(w_{1}+w_{2},\ldots ,+\ldots w_{n}\right)},$$

where $B^{t}$ is the buffer occupancy ratio at time *t*, and is defined as

(3) $$B^{t}=\frac{B_{cur}}{B_{max}},$$

where $B_{cur}$ is the current buffer size at time *t*, and $B_{max}$ is the maximum buffer size.

#### 3.2.4. Short-Term Power Consumption Ratio

In general, it is difficult to replace the battery of the sensors deployed in the wild field. If the energy of a specific node is quickly exhausted, it leads to a decrease in the network lifetime. To improve the overall network lifetime, we adopt the power consumption ratio as a decision variable to select a parent node. The power consumption ratio (*E*) can be calculated as,

(4) $$E=\frac{\left(\alpha -1\right)T_{cu}*Idle_{e}+\left(\alpha \right)L*Tx_{e}+\left(\alpha *D\right)Rx_{e}+Sleep_{e}}{E_{total}},$$

where $T_{cu}$ denotes the cumulative delay due to backoff or retransmissions. α is the number of backoffs or frame retransmissions. *L* denotes the payload length. $Idle_{e}$, $Tx_{e}$, $Rx_{e}$, and $Sleep_{e}$ are the amount of energy consumption for each transceiver mode, and $E_{total}$ is the total amount of energy.

### 3.3. Problem Statement

To jointly achieve multiple objectives, we aim to find an optimal parent node according to changes in network conditions. For this, we consider multiple decision criteria; however, they are in a trade-off relationship. Hence, we aim to provide a reasonable balance between conflicting goals (i.e., high reliability, low latency, and energy efficiency) rather than jointly achieving them. In our previous approach [19], each node calculates the weighted sum of multiple criteria for each neighbor, and then chooses the node with the highest weighted value. It shows reasonable trade-offs between the performance metrics; however, the decision maker must calculate the weight factors based on its preference or background knowledge of the network. By contrast, in this work, RL is used to enable each node to utilize empirical data about the network so that the parent node can be adaptively selected according to the network state.

## 4. Proposed Tree Routing Protocol

**Figure 2:**
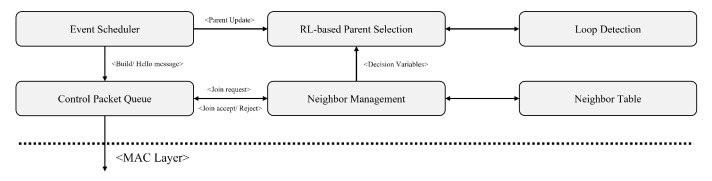
Proposed protocol architecture.

As shown in Figure 2, the proposed tree routing protocol consists of several components. Specifically, each component interacts with others to build a tree topology and finds an optimal routing path via a parent selection. In this section, we first describe the basic operations of the proposed routing protocol and then specify the RL-based parent selection algorithm.

### 4.1. Tree Construction

**Figure 3:**
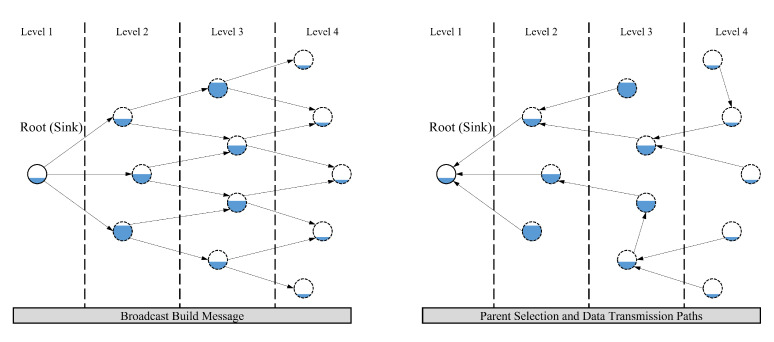
An illustration of tree-based routing.

As illustrated in Figure 3, the sink node periodically broadcasts a build message to form a tree structure. The build message includes the number of hops to the sink node (i.e., hop count). When a node receives a build message, it stores the hop count in the neighbor table and increments the hop count in the build message by one. Then, the node rebroadcasts the build message. Since a node chooses a neighboring node with the same or fewer hops as a parent node, the hierarchical level of nodes is autonomously determined based on the number of hops to the sink node. In addition, each node periodically broadcasts a hello message containing the proposed decision criteria (i.e., *P*, *B*, and *E*). Upon receiving the hello messages from the neighbors, each node stores the decision criteria in the neighbor table. These variables are used to choose a parent at each node. The details of the tree construction are given in Algorithm 1.
**Algorithm 1** Tree initialization at node *k*.**Initialization:**
  $S_{id}$: The ID of sink node

  $H_{i}$: The number of hops to the sink node at sensor node *i*

  NT: Neighbor table
**Algorithm:**
1: **if** 
node_type=root 
**then**

2:    **periodically** broadcast a build message $B_{msg}$

3: **else**

4:    **periodically** broadcast a hello message $H_{msg}$

5: **end if**

6: **upon** receiving $B_{msg}$ from node *i*

7:   Update $S_{id}$ and $H_{i}$ of $B_{msg}$ in NT

8:   $H_{i}$ in $B_{msg}\leftarrow$$H_{i}$ in $B_{msg}+1$

9:   Broadcast $B_{msg}$

10:   Call ***Parent_Selection ()***


### 4.2. Parent Selection

**Figure 4:**
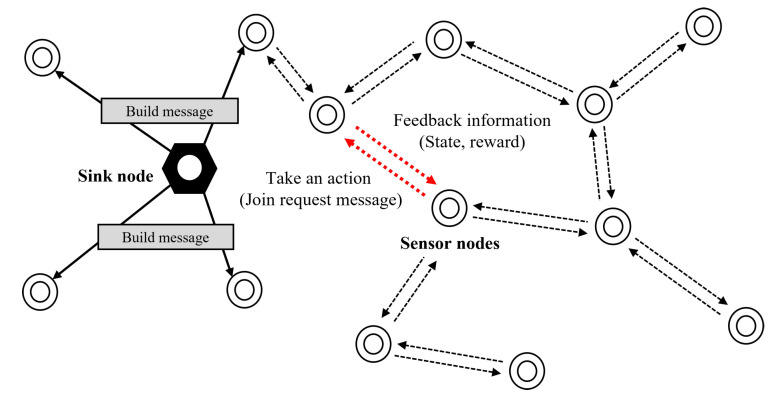
An example: RL-based parent selection.

To provide a reasonable trade-off between multiple objectives, we aim for the node to adaptively change its parent node according to network conditions. As illustrated in Figure 2 and Figure 4, each node recognizes the current network state using the proposed cognitive metrics, and then finds an optimal parent node based on empirical data about the network. To this end, we propose an RL-based parent selection algorithm. In this subsection, we first define the state space, action set, and reward function using several cognitive metrics, and then specify the RL-based parent selection algorithm.

#### 4.2.1. RL Model

Here, we define the state, action, and reward function as follows.

- State: The state space can be defined as a set of three decision criteria, denoted by $s={\left\{P_{i},B_{i},E_{i}\right\}}_{i\in N}$. That is, each node (i.e., agent) selects a parent node in consideration of the link quality, congestion level, and remaining energy of the neighboring node.
- Action: The action space is defined as a set of candidate parent nodes in the neighbor table. It should be noted that the set of candidate parent nodes only contains the neighboring nodes with the same or fewer hops to the sink node than itself.
- Reward: Our approach to define the reward function is that if the agent selects a node as a parent node and that action increases the frame retransmission, packet error rate, and energy consumption, then the agent obtains a lower reward. Otherwise, the agent gets a high reward.

#### 4.2.2. Proposed Algorithm

To find the best parent node, we define multiple cognitive metrics (i.e., hop count, received signal strength, buffer occupancy ratio, and power consumption ratio). Our key idea is that each node adaptively changes its parent node according to the current network state using the cognitive metrics. Obviously, the more cognitive metrics we consider, the more benefits achieved when choosing the parent node; however, complex decision-making problems may arise. To solve this problem, in this work, we present the RL-based parent selection algorithm.

The proposed algorithm selects the node with the highest reward as a parent node. Algorithm 2 shows the detailed process of the parent selection. Given a state *s*, each node chooses a parent node among the neighboring nodes based on epsilon-greedy algorithm (lines 4–5). When the parent node selection is complete, the node observes the reward *r* and new state $s^{\prime }$ from the network environment (lines 6–8). The node then sends a join request to the candidate parent node (line 9). After receiving the join request message, the selected parent node replies to the corresponding node with a join acceptance message or a join rejection message (lines 12–18). Until the episode ends, each node finds an optimal parent node through trial and error.

Recall that the node selects the neighboring node with the same or fewer number of hops to the sink node than itself as a parent node; thus, the hierarchical level of nodes is autonomously configured based on the number of hops. However, each node can select a neighbor node in the same level (i.e., a node with the same number of hops to the sink node) as a parent node. As a result, a cycle can be generated. To check the cycle between the parent and child nodes, each node sends a join request message to the candidate parent node with its list of child nodes (line 9). The node receiving the join request message detects a loop based on the list of child nodes (lines 19–24). If a loop is not detected, the node replies a join acceptance message. Otherwise, it returns a join rejection message.
**Algorithm 2** Parent selection at node *k*.**Initialization:**
   $N_{child}$: List of child nodes

   PID←⌀ // The ID of parent node

   Initialize Q(s,a) arbitrarily
**Algorithm:**
1: **for** each episode **do**

2:    Initialize *s*

3:    **for** each step of episode **do**

4:      Choose *a* from *s* using policy derived from *Q*

5:      PID←a

6:      Observe *r*, $s^{\prime }$

7:      $Q\left(s,a\right)\leftarrow Q\left(s,a\right)+\alpha [r+\gamma max_{a^{\prime }}Q\left(s^{\prime },a^{\prime }\right)-Q\left(s,a\right)]$

8:      $s\leftarrow s^{\prime }$

9:      Send a join request message $J_{request}$ with $N_{child}$ to the selected parent node (PID)

10:    **end for**

11: **end for**

12: **upon** receiving a join request message $J_{request}$ from node *i*

13:   r←  
***Loop_Detection***
 
**($N_{child}$ of $J_{request}$)**

14: **if**  
r=True
 
**then**

15:    Send a join rejection message $J_{reject}$ to node *i*

16: **else**

17:    Send a join acceptance message $J_{accept}$ to node *i*

18: **end if**
**Function: Loop_Detection** (*N_child_*)

19: **for** each node *i* in $N_{child}$ **do**

20:    **if** my_id=i **then**

21:      return True

23:    **end if**

24: **end for**

24: return False


### 4.3. Rate of Parent Change

Each node should update its parent node periodically because the network condition frequently changes in WSNs. For example, if the parent node is not updated for a long period of time, an excessive load occurs on a specific node. To achieve a stable topology, each node periodically updates its parent node based on hello message interval. However, if the node frequently changes the parent node, an additional overhead may occur. In the next section, we present the effect of the rate of parent change on the network performance.

## 5. Performance Evaluation

### 5.1. Simulation Setup

In this work, we simulate the proposed scheme using the OPNET modeler version 18.7. The simulation parameters are given in Table 1. We conducted the simulation 100 times with a 95% confidence interval. Specifically, we run the simulations for 3600 s and set the network size to 5000 m × 5000 m. To build a large-scale network, we set the number of sensor nodes to 100 and the number of sink nodes to 5. To build a tree topology, the sink node periodically broadcasts a build message every 20 s. After receiving the build message, each node updates the number of hops to the sink node in the neighbor table and start to choose a parent node. In addition, all nodes periodically send a hello message to its neighbors every 5 s. We set the sizes of the build and hello messages to 192 and 128 bits, respectively. To verify that the proposed scheme provides a reasonable trade-off between multiple objectives by adaptively changing the parent node according to changes in network conditions, we observe the performance metrics by varying the traffic bit rate and bit error rate. To simulate congestion, we change the traffic bit rate from 1000 to 5000 bits/s. In addition, we change the bit error rate from $10^{-2}$ to $10^{-6}$ in order to make the link condition dynamic.

We compare the performance of the proposed scheme with the linear weighted sum-based parent selection algorithm [17] and the MCMD-based parent selection algorithm. The linear weighted sum-based scheme considers the number of hops, buffer occupancy ratio, link quality, and residual energy as decision metrics. Here, the weight of each metric is fixed at the same ratio. On the other hand, the MCDM-based parent selection scheme logically determines the relative importance between the decision metrics based on the preferences or background knowledges of the decision maker. Specifically, in our previous work, we (i.e., the decision maker) set the relative importance of each metric based on the following rules. When the network is unstable, we increase the weight of the received signal strength, leading each node to choose a parent node with good link conditions. If the link conditions of the candidate nodes are not significantly different, each node changes another node with a low buffer occupancy and high residual energy to a parent node.

### 5.2. Reliability

**Figure 5:**
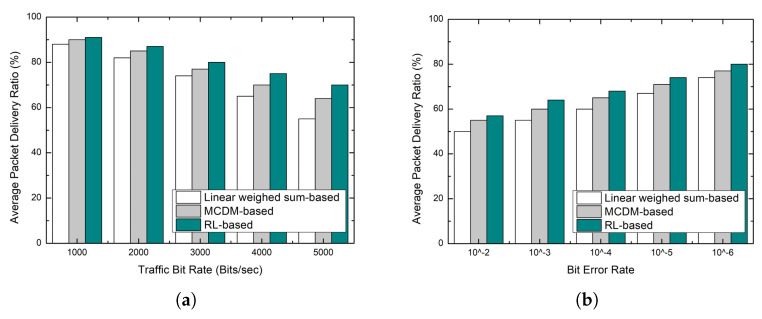
Simulation results: packet delivery ratio. (**a**) Packet delivery ratio according to traffic bit rate; (**b**) Packet delivery ratio according to bit error rate.

The main factors affecting the packet delivery ratio are buffer overflows caused by congestion and bit errors according to the link conditions. As shown in Figure 5, all schemes cause packet loss owing to congestion as the traffic bit rate increases. The linear weighted sum-based scheme considers link quality in the decision-making process. However, since the weights among the multiple metrics are equal, the parent node is rarely changed even if a buffer overflow occurs. On the other hand, since the MCMD-based scheme has a higher weight of the buffer occupancy ratio, packet loss owing to congestion is less than that of the linear weighted sum-based scheme. In addition, it adaptively selects a node with good link quality as the bit error rate increases, thus showing a better packet forwarding rate.

However, in the MCDM-based scheme, the weights between metrics cannot be changed at runtime; thus, it has a clear limitation in that it is difficult to respond to changes in network scenarios. In contrast, the proposed scheme shows the best performance because it learns how to cope with changes in network conditions at runtime through trial and error.

### 5.3. Latency

**Figure 6:**
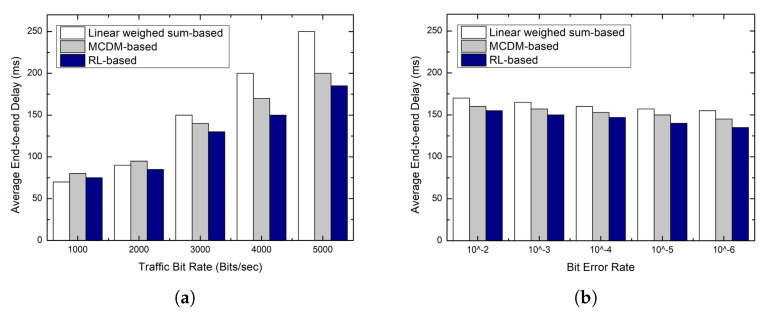
Simulation results: end-to-end delay. (**a**) End-to-end delay according to traffic bit rate; (**b**) End-to-end delay according to bit error rate.

The main factors affecting the end-to-end delay in tree routing are the number of hops to the sink node and the queuing delay. The traditional tree routing chooses a parent node based on the number of hops to the sink node. This shows the best performance when the traffic bit rate is low. However, as the traffic bit rate increases, congestion occurs at a node with a small number of hops to the sink node, resulting in a sharp increase in end-to-end delay. To solve this problem, the linear weighted sum-based scheme considers the congestion metric with the number of hops in the decision-making process. As shown in Figure 6, since the weights between the metrics are the same in the linear weighted sum-based scheme, load distribution is not effectively performed as the traffic bit rate increases. This increases the queuing delay and thus it shows the worst performance. In addition, when the bit error rate increases, a node having a better link quality rather than having a lower number of hops can be selected as a parent node; hence, the end-to-end delay is slightly increased. In the MCMD-based scheme, load balancing is performed properly because the decision maker pre-determines the weight of the congestion metric by considering the network environment. However, when the bit error rate increases, a node with a better link quality rather than with a lower number of hops is selected as a parent node; hence, the end-to-end delay is slightly increased.

As described above, in the MCMD-based scheme, the decision maker pre-determines the weight of each metric based on prior knowledge of the network environment. Thus, it performs well for pre-configured network scenarios, but cannot cope with changes in network conditions that occur at runtime. In contrast, in the proposed scheme, each node learns the optimal action according to the network environment through Q-learning. The proposed scheme shows the best performance because it can adaptively cope with changes in network conditions.

### 5.4. Energy Efficiency

The main factor that increases average power consumption in tree routing is frame retransmissions owing to packet loss caused by congestion and link breakage. In addition to energy efficiency, in tree routing, it is necessary to balance the energy consumption between sensor nodes to ensure the diversity of routing paths.

**Figure 7:**
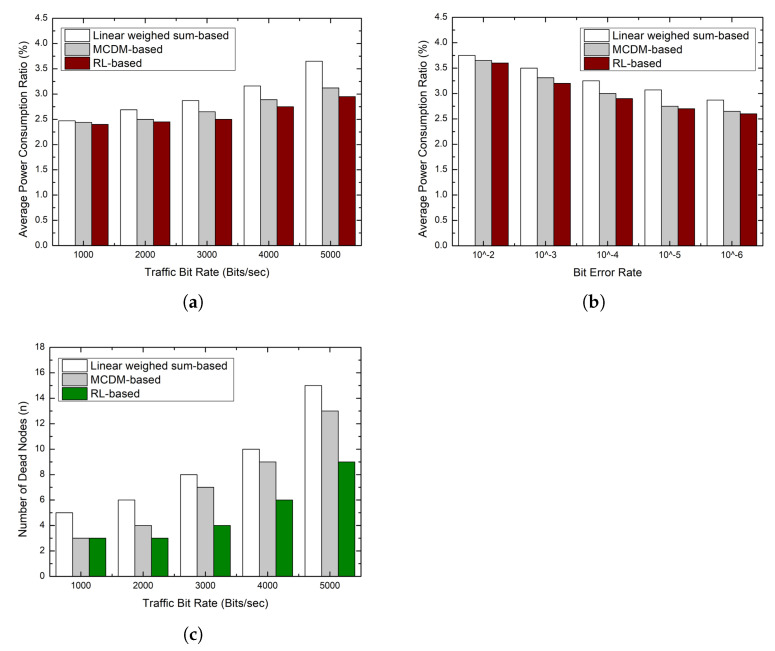
Simulation results: power consumption ratio. (**a**) Power consumption ratio according to traffic bit rate; (**b**) Power consumption ratio according to bit error rate; (**c**) Number of dead nodes according to traffic bit rate.

The traditional tree routing selects a parent node based on the number of hops to the sink node; thus, the packet loss significantly occurs owing to congestion as the traffic bit rate increases. As a result, additional energy consumption increases owing to frame retransmissions. In addition, when the link quality is bad, the number of retransmissions also increases. To address this problem, the linear weighted sum-based scheme considers the link quality and buffer occupancy ratio together in the decision-making process, but it is difficult to respond to changes in network conditions because the weights between multiple metrics are not properly adjusted. As a result, as shown in Figure 7, the energy consumption per unit time is the highest, and the number of dead nodes is the highest.

The MCMD-based scheme shows better performance than the linear weighted sum-based scheme because the decision maker knows the network environment in advance and determines appropriate weights accordingly. However, as previously analyzed, it is not possible to adjust the weights of metrics at runtime. By contrast, the proposed scheme recognizes the network environment through various cognitive metrics and finds the best policy to optimize the performance metrics through trial and error. As a result, the proposed scheme shows better performance than the MCDM-based parent selection scheme.

### 5.5. Discussion

As the traditional tree routing selects a parent node based on the number of hops to the destination, it cannot cope with various problems, such as link breakage and congestion. To address this problem, the linear weighted sum-based scheme jointly considers multiple cognitive metrics, such as the hop count, link quality, buffer capacity, and residual energy in the decision-making process. However, in the process of deriving the weighted sum, the weights between the multiple metrics are determined to be the same; thus, it cannot cope with changes in network conditions.

To solve this problem, the MCDM-based scheme proposed in our previous study aims to respond to changes in network environment by logically determining the weights between multiple metrics. However, as the decision maker pre-determines the weights of each metric based on prior knowledge, there is a clear limitation in that the weights cannot be changed at runtime. To overcome this limitation, the proposed scheme recognizes the network environment using multiple cognitive metrics and finds the optimal policy to jointly optimize the performance metrics through Q-learning. In addition, we use WMA to prevent sudden changes in cognitive metrics. As a result, the proposed scheme provides a flexible trade-off between conflicting performance metrics by adaptively changing the parent node according to changes in network conditions.

## 6. Conclusions

To jointly achieve multiple objectives in tree routing, we revisit the classic problem of finding an optimal parent node in a complex deployment scenario. Our key idea is to find the best parent node by utilizing empirical data about the target network via Q-learning. Specifically, our contributions are: (1) We propose three types of cognitive metrics to cope with various network scenarios. (2) We present a system model for applying RL in tree routing and specify concrete algorithms regarding the tree construnction, loop detection, and parent update. (3) We define a state space, action set, and reward function to find the best parent node via Q-learning. Through comprehensive simulations, we demonstrate that the proposed parent selection scheme can strike a balance between the performance metrics regarding end-to-end delay, packet delivery ratio, and energy consumption.

## Figures and Tables

**Table 1:** Simulation parameters.

Parameter	Value
Number of sensor nodes	100
Number of sink nodes	5
Network size	5000 m × 5000 m
Simulation time	3600 s
Transmission range	300 m
Data processing rate	15,000 bits/s
Buffer size	100,000 bits
Traffic model	IP traffic flow
Traffic bit rate	1000 to 5000 bits/s
Bit error rate	$10^{-2}$ to $10^{-6}$
MAC	802.11
Build message interval	20 s
Hello message interval	5 s
Maximum number of episodes	4000
Maximum number of steps	300
Minimum epsilon/epsilon	0.1/1.0
Exploration ratio	0.5
Reward discount factor	0.99

## Data Availability

Available on request.
